# A Case of Obstinate Hiccough and Its Successful Treatment

**Published:** 1901-10

**Authors:** J. A. Durrent

**Affiliations:** Marysville, Wash.


### CLINICAL REPORT.

### A Case of Obstinate Hiccough and its Successful Treatment.

By J. A. DURRENT, M. D., Marysville, Wash.

ON MAY 16, I was called to see Mr. S., aged 41, married, the father of three healthy children, whose family history was negative.

As to his manner of life, habits, and the like, nothing was elicited except that he had very peculiar ideas about his diet. Some very wholesome and easily digested foods he would not use, while he was fond of others that are very hard to digest. Two years previous to this attack the patient had a similar experience with hiccough that lasted three days. During that attack the hiccough was not severe, and did not prevent him from following his usual occupation, beyond the embarrassment that it caused. I found that his stomach had given rise to some trouble for years. Very little definite data could be obtained on this point, but the symptoms suggested chronic gastritis. The patient always had an abnormal craving for sodium chloride. For the last two years he had not complained very much of his stomach.

At the time I was called the man was suffering from pain in his back and lower extremities, and a headache of moderate severity. His tongue was slightly coated and his temperature was 101.8°. The pulse was good and a little above normal. I put him upon a liquid diet and ordered him to remain in bed, besides prescribing calomel, phenacetin and Dover's powder.

The next day all the symptoms had abated and the patient was feeling quite comfortable, although he had commenced to hiccough occasionally during the day. At this time the hiccough could be stopped by simple means, such as traction upon the tongue.

The following day, May 18, the hiccough had become worse and began to alarm the family. Small doses of calomel were given without any result. Amyl nitrite only stopped it for a few minutes and finally neither it nor nitroglycerin were of any benefit. By Sunday the attacks had become so severe that they continued steadily all day, unless relieved by partial anesthesia. The hiccough was so violent that the bed would shake with the movement. During the day, I used large doses of chloral and potassium bromide, per rectum, and atropin and morphia hypodermically. Even when the patient was completely under the influence of these drugs it would be necessary occasionally to administer chloroform to stop the hiccoughs. Finally, they became so persistent as to occur when the patient was totally unconscious.

Monday morning I washed out the patient's stomach quite thoroughly, affording relief for an hour. This operation was repeated daily for three days. At this stage I called in two consulting physicians, but neither of them suggested a different course of treatment. However, on Monday afternoon and Tuesday, I persevered with traction on the tongue; I observed also that pressure on the hyoid bone would at times stop the hiccough.

On Wednesday, about noon, I telephoned Dr. Howard, asking him if he could suggest any other remedy that might prove helpful. He replied that I might give chloretone a trial, which I did. On Wednesday afternoon, I prescribed twenty grains of chloretone, the dose to be repeated every three hours until two drachms had been taken. As a result the hiccough stopped for intervals of two and three hours, during Wednesday evening, and by Thursday morning when anesthesia was complete it had ceased entirely, and has never returned except once about a week later; it lasted then for only a few minutes and stopped without treatment.

The patient remained in a comatose condition until Sunday, the 26th, when he became partly conscious; on Monday his mind was clear at intervals and by Tuesday he had recovered complete consciousness. During this comatose condition feces and urine passed involuntarily, and the patient was fed on milk, with eggs, by means of a stomach tube. The pulse at first ranged from 110 to 120, and was quite weak, although regular. The respirations ranged from 40 to 50, and the temperature from 101° in the morning to 103° in the evening. By Sunday, May 19, the pulse rate had fallen to 100 and there was much improvement in its quality. The fever was less and by the middle of the week the temperature was normal, while the respirations had dropped to 35. During this time the medicinal treatment consisted of digitalis, whiskey and strychnin.

From this time on recovery was uneventful. The man has complained of nothing, except two blisters that were placed on his abdomen in the hope of doing some good, and a swelling in his left calf, which has nearly disappeared.

In conclusion it might be of interest to name the various remedies and means that had been used to stop the hiccough; these were calomel, amyl nitrite, nitroglycerin, valerian, chloral, potassium bromide, atropin, morphin, chloroform, oil amber, hydrochloric acid, pepsin, cerium oxalate and bismuth. Also lavage, traction on the tongue, pressure on the base of the tongue, pressure over the phrenic nerve, holding of the hyoid bone, blisters over the exit of the lower four cervical nerves, blisters on the abdomen, and futile attempts to cause sneezing. In addition to these, pillows were placed under the patient's back, so that he assumed the position of one in a state of opisthotonos.

An examination of the lungs, heart and urine gives us no light on the cause of the trouble in this case. The stomach washings at first were of an olive green color, but after the first day were very clear. I am still of the opinion that the hiccough was caused by some condition in the stomach,and feel that the anesthetic and hypnotic effect of the chloretone were instrumental in controlling the reflex action.

